# Unreliable Medical Certificates

**Published:** 1898-10-22

**Authors:** 


### UNRELIABLE MEDICAL CERTIFICATES.

We have again and again insisted upon the importance of medical men taking due heed before signing medical certificates. That sufficient care is not always taken is, we think, pretty obvious. A preparatory schoolmaster writes to the effect that, out of 32 boys whose certificates he holds, only 14 had been examined on their chest, 17 in the throat, several had simply been told to put their tongues out, five had not been examined either as to tongue, throat, or chest, and none at all were examined on the head. "The net result is," he says, "that, out of the 23 medical men from various parts of England and Scotland, only seven, including two distinguished London doctors, made a thorough examination before signing this explicit declaration—'I have carefully examined.'" The form of the certificate in question is a very proper one, and one which ought to ensure a proper examination being made before it is signed by anyone, however intimate his knowledge of the boy in question. It runs as follows: "I certify that I have this day carefully examined ———, who returns to school to-morrow, and that there are no signs of any infectious complaint about him." It is difficult to understand what could induce any man to put his name to such a form as this without having made a careful and pretty complete examination. On the other hand it must be understood that the making of such an examination is a matter requiring the exercise of a high degree of professional skill and deserving of a proper fee, and although we do not hold that the poverty of the fee is any excuse for giving a certificate on imperfect grounds, we think it extremely likely that a good deal of the laxity which has crept into the giving of medical certificates is due to the unwillingness of the public to pay properly for a proper article. Clubs, school boards, friendly societies, convalescent homes, charitable associations of all kinds, and many employers of labour, all expect to be furnished with medical certificates of one form or another; but not one of them expects to pay. Yet to refuse such certificates is often to impose a great hardship upon one's patients, and thus has grown up a bad and rotten system. So far indeed has the evil gone that many of the people who so readily ask for and expect to receive gratuitous certificates are so bold in their demands that they print their own forms and decline to receive certificates in any other shape; and, unfortunately, in these forms they insert items in regard to which it is absolutely impossible for the medical man to certify of his own knowledge. Even this preparatory schoolmaster, who complains that he lost £500 in consequence of a boy bringing back to school the infection of scarlet fever, had been in the habit of demanding a certificate which had this very vice. It ran as follows: "I certify that ——— is quite fit to return to school, that he has not had any infectious illness during the holidays, &c." That an educated man, who no doubt teaches his boys logic, should accept and even ask for such a form of certificate, certifying as it does to a fact which in nine cases out of ten the doctor can only know by hearsay, is enough of itself to show how lax is the public feeling in regard to this matter. How can the medical man who is expected to sign this certificate know, except from mere hearsay, what has happened to the boy during the seven or eight weeks that he has been visiting all over the country? The fact is that the schoolmaster wants the certificate, not only for the sake of keeping fever out of the school, but for his own protection, so that he may wave it in the face of indignant parents in case fever should creep into his establishment; so he words it as stringently as possible with the object of throwing all responsibility on the doctor, who, poor man, finds himself in a corner—for how can he refuse to certify without openly flouting the veracity of the lordly parent who assures him that the boy has been well all the time? And so the game goes on, and medical certificates are dragged in the mud. We have not a word to say in defence of what we fear may without untruthfulness be called "the present system," but we feel pretty confident that the very regrettable state of affairs which has now been reached is largely due on the one hand to the enormous multiplication of the demands for gratuitous certificates, and on the other hand to the growing practice of asking for such certificates on printed forms containing various extravagant assertions. Certain School Boards, for example, have not only asked medical men to certify to the fact that a child is unfit to attend school, but have so worded their form of certificate that the medical man signing it has to certify to the time when the child will probably be able to return to school! Again, in the certificate forms which are now in use in London under the Public Health Act, the following words occur: "I further certify the following particulars required under the said section to be given," and then come various particulars, among which is this item: "If the patient is an inmate of a hospital —(a) Place from which the patient was brought to the hospital." How does a medical man sitting among his out-patients or seeing his patients in the wards know, except by hearsay, where a child comes from? He cannot know; and yet in the form of certificate issued by the vestries in accordance with Act of Parliament, a form of certificate which he is compelled to sign, he is made to certify to what he cannot know. This may seem a small matter, but it is by the repetition of such little perversions of truth, done day after day under the sanction of public authority, that conscience in such matters gradually becomes dulled. If medical men would refuse to certify to anything that they do not know of their own knowledge, a medical certificate would come to take its proper place as a responsible document. But what a clearance there would be among the certificates!

